# Custom-made 3D printing-based cranioplasty using a silicone mould and PMMA

**DOI:** 10.1038/s41598-023-38772-9

**Published:** 2023-07-25

**Authors:** Loránd Csámer, Zoltán Csernátony, László Novák, Viktor Zsolt Kővári, Ágnes Éva Kovács, Hajnalka Soósné Horváth, Sándor Manó

**Affiliations:** 1 Department of Orthopaedic Surgery, Faculty of Medicine, University of Debrecen, Debrecen, Hungary; 2 Department of Neurosurgery, Health Centre of Hungarian Army, Budapest, Hungary; 3 Department of Neurosurgery, Faculty of Medicine, University of Debrecen, Debrecen, Hungary

**Keywords:** Biomedical engineering, Neurology, Fracture repair

## Abstract

All types of cranioplasty techniques restore the morphology of the skull and affect patient aesthetics. Safe and easy techniques are required to enhance patients’ recovery and the rehabilitation process. We propose a new method of cranioplasty. The 3-dimensional (3D) reconstruction of a thin-layer computed tomography (CT) scan of the skull was used to reflect the intact side onto the defect and subtract the overlapping points from one another. In this way, a 3D model of the planned implant can be built in the required shape and size. The precise fit of the implant can be checked by printing the defective part of the skull in case it can be modified. A sterilisable silicone mould based on the finalized model was created afterwards. Polymethyl methacrylate implants were prepared directly in an aseptic environment in the operating room during surgery. Between 2005 and 2020, we performed 54 cranioplasties on 52 patients whose craniotomies were performed previously for indications of traumatic brain injury, stroke or tumour surgeries. No technical problems were noted during the operations. In 2 cases, septic complications that occurred were not connected to the technique itself, and the implants were removed and later replaced. Our proposed technique based on 3D-printed individual silicone moulds is a reliable, safe, easily reproducible and low-cost method to repair different skull defects.

## Introduction

Various techniques have been developed to repair and precisely reconstruct skull defects. The challenges of these techniques are based on individual cases, and the institutionally developed methods can be insufficient for certain patients^1–3^. Manual moulding with polymethyl methacrylate (PMMA) is the simplest method to cover defects. This method is still applicable to smaller defects with nearly even surfaces at easily reached locations. To cover larger surfaces, different titanium implants can be used, but the complication rate in the long term can obscure the indication in cases where plastic reconstruction of the skin is needed.

The increase in availability of 3-dimensional (3D) printing was a turning point in the area of individual osteoplasty and particularly for cranioplasty^4–6^. Operations after traumatic brain injury and the widespread use of decompression in stroke patients have increased the demand for cranioplasty. Easy, surgeon-friendly, reproducible and low-cost techniques are necessary to develop. A cranioplasty procedure based on a special 3D printing method, well suited even for complicated geometries, has been in use at the University of Debrecen since 2005. In this procedure, first we print a sample that matches the shape and size of the intended replacement; then, based on this cast, we produce a silicone mould that in turn can be used to fabricate a replacement made of PMMA during surgery.

## Methods and materials

We performed 54 cranioplasties on 52 patients. The male/female ratio was 2.46, and the mean age was 40.2 years (SD ± 13.41). The youngest patient was 17 years old at the time of cranioplasty while the oldest was 65 years old. The mean implant volume was 52.19 cm^3^ (SD ± 27.37). The mean implant surface area was 218.8 cm^2^ (SD ± 91.04). All of the operations were performed 3 months after craniectomy. All methods were performed in accordance with the relevant guidelines and regulations.

**Figure 1:**
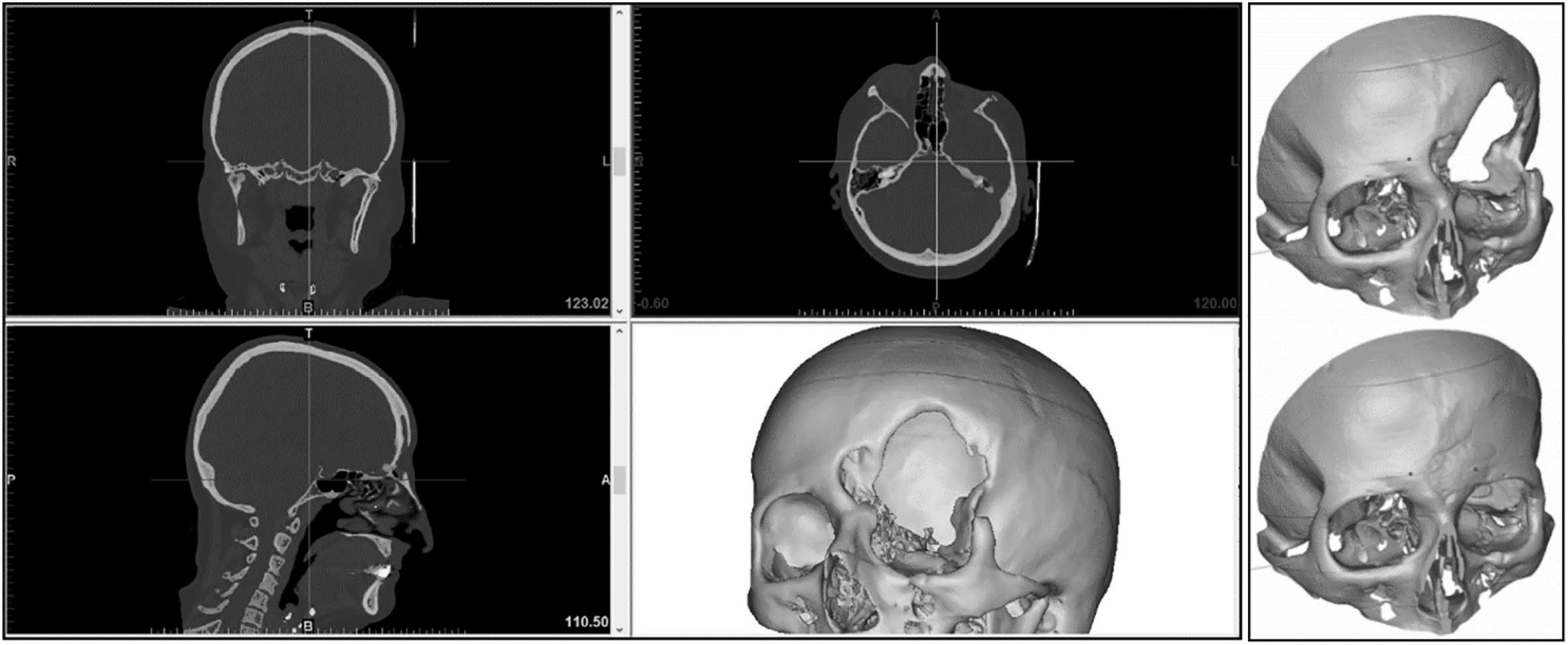
3D reconstruction based on CT scans and the design process using the mirroring technique. Image was created by the authors with Materialise Mimics 21.0 software (URL: https://www.materialise.com/en/healthcare/mimics-innovation-suite).

**Figure 2:**
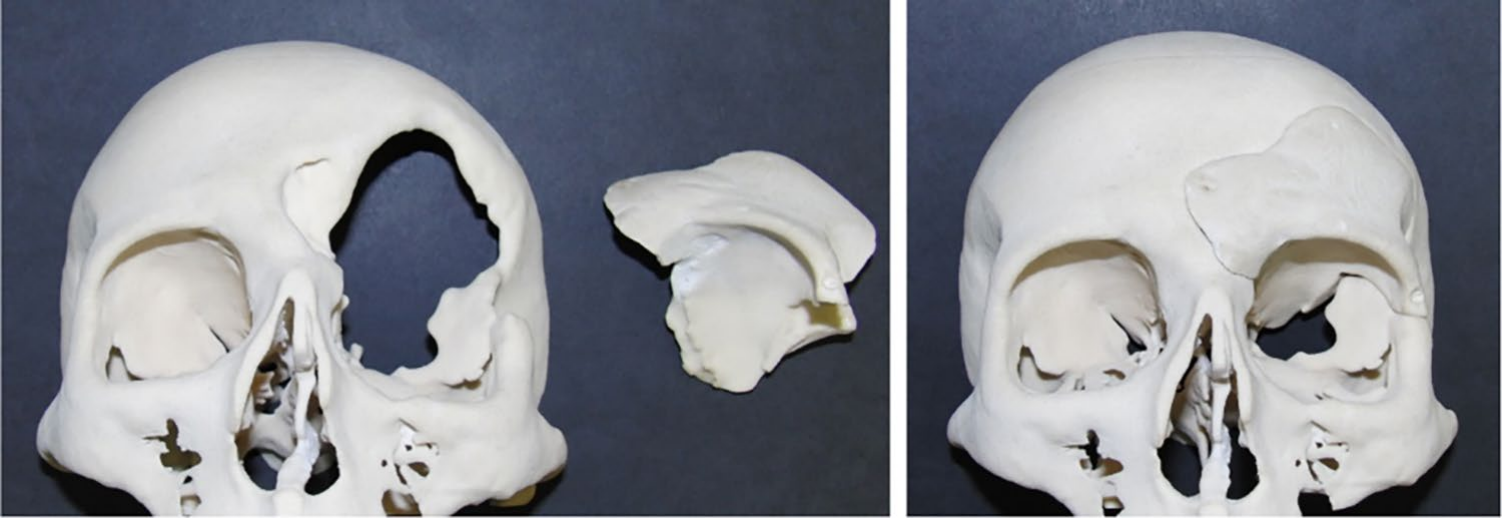
The 3D-printed skull segment and replacement. Photos were taken by the authors.

**Figure 3:**
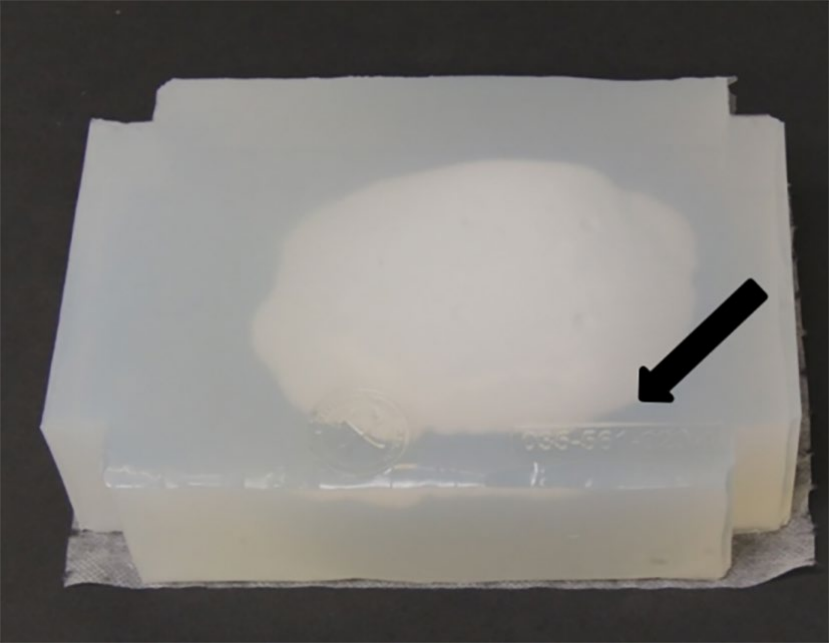
The silicone mould with the identification number. Photos were taken by the authors.

**Figure 4:**
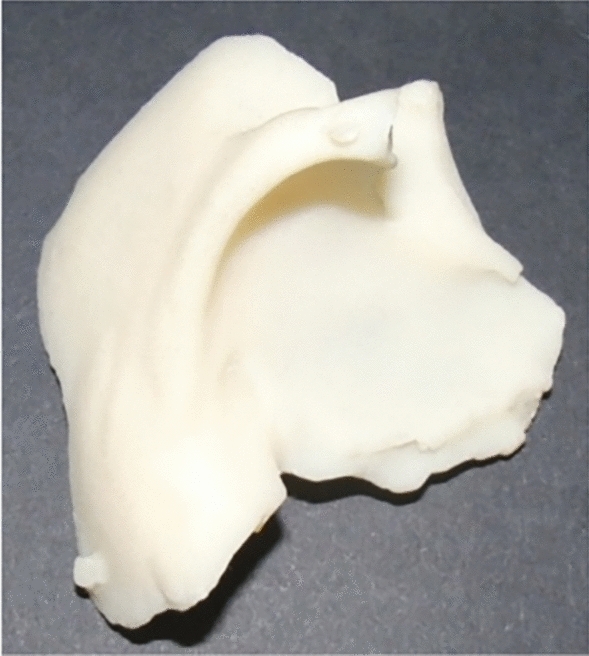
The replacement made with PMMA. Photos were taken by the authors.

**Figure 5:**
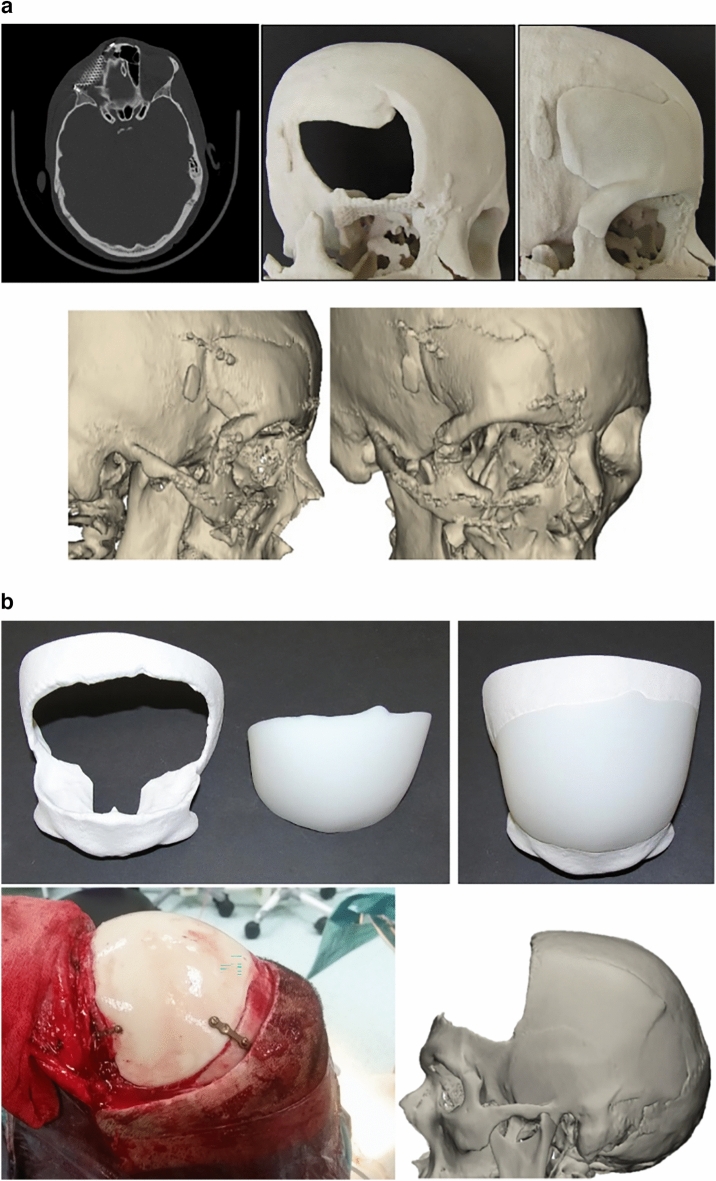
a and b Preoperative and postoperative pictures from different patients treated using our method. Photos were taken by the authors. Image was created by the authors with Materialise Mimics 21.0 software (URL: https://www.materialise.com/en/healthcare/mimics-innovation-suite).

When cranioplasty was indicated, high-resolution CT scans were obtained with a slice thickness of 1 mm. The 3D reconstruction from DICOM files was performed with the Mimics® (Materialise, Belgium) software system. As a next step, a geometric form was generated that precisely fit the defect area and reproduced the original contours. This was mostly done based on the symmetry of the skull by mirroring its intact half through the midsagittal plane. To minimize the time needed for the calculations, we removed those parts of the models that are not essential according to the case (Fig. 1). The form of the implant of the cranioplasty was obtained by Boolean subtraction of the model with the defect from the reflected model representing the intact skull. If the defect cuts the symmetry plane or the replacement cannot be generated by mirroring due to other circumstances, consultation with the neurosurgeon facilitated the planning process. In 2 cases, when bilateral fronto-temporo-basal and bifronto-temporo-occipital decompression had to be reconstructed, the precompression CT scans were used in the planning process. Not only the replacement but also the defective part can be printed out to check the perfect fit before surgery. Printing was performed on Connex 260 (Stratasys, USA) equipment utilizing Objet technology. If necessary, the printed model can be fitted even more precisely by cutting, milling and grinding (Fig. 2). It is also possible to add blind holes that can lead during the operation the drill if needed. After finalizing the shape of the model, we fabricated a silicone mould using Protosil RTV 245 (Antropol, Germany), a two-component silicone material that becomes biologically inert after solidification. The mould is transparent, heat resistant up to 200 °C, and easily sterilisable; in addition, solidified PMMA does not adhere to it. Considering the hardness of the silicone 40A Shore used, the thickness of the silicon should be at least 12 mm surrounding the sample to avoid deformation while forming the low-viscosity bone cement mixture into the mould. After moulding, the silicone is kept at a temperature of 50 °C for 12 h to achieve complete solidification. All the moulds that conform to the General Data Protection Regulation (GDPR) (Fig. 3) are labelled. Through the lateral cut, the model of the defect is removed. The cut allows the silicone mould to be opened and closed like a book, paying special attention to a precise fit. To validate the process, PMMA is formed into the mould and inserted into the previously printed model containing the defect. No macroscopical gaps were observed in either case. The final implant is created at the operation theatre under sterile circumstances. PMMA is formed into the mould (Fig. 4). The forming of the bone cement must begin immediately after proper mixing to ensure that the viscosity is low enough to avoid any deformity. During the moulding process, special attention must be paid to the uniform distribution of the cement in the mould. The formation of air bubble inclusions can be prevented by using the proper technique. After polymerization for at least 10 min, the mould is opened through a lateral cut, and the implant is easily removed without any adhesion to silicone. As the last step of the procedure, the implant is fixed to the nearby bones with small plates or transosseous sutures through small bur holes. The main steps of the procedure are presented on Fig. 5.

### Ethics approval

The experimental protocol was approved by the Regional and Institutional Ethics Committee, Clinical Center of the University of Debrecen (DE RKEB.IKEB 6371-2023).

### Informed consent

Informed consent was obtained from all individual participants included in the study at the moment of hospitalization for surgery.

## Results

In all cases, the technique was successfully used during the operations. Thirty-four implantations were performed poststroke, 4 were performed after tumour removal and 16 were performed after traumatic brain injury. Two patients had bilateral implants, one of them was composed of one piece after bifronto-temporo-basal craniectomy. Two patients from the trauma group underwent 2 operations because of wound infections that necessitated the removal of the implant. In these 2 cases, the same silicone mould was used to create the implant 3 months later (Table 1). The fittings were adequate, and postoperative cosmetics were comforting in all cases. In one patient where part of the frontal cranial base and frontal sinus had to be reconstructed after several years of open craniocerebral injury, a short period of epidural pneumocephalus developed after 2 weeks when he blew his nose. The air was absorbed within days, but another event occurred after another 3 weeks with a similar course. The reported complications were not related to the technology itself.

**Table 1:** Data of the patients treated by our newly developed method.

Age	Sex	Vol cm^3^	Area cm^2^	Comp			
49	Female	58.2	270	–			
32	Male	45.7	271.5	–			
39	Male	48.3	234.1	–			
27	Female	18.6	107.1	–			
60	Male	52.4	252.7	–			
58	Female	108.3	355.4	–			
37	Male	16.1	80.6	–			
54	Female	35.7	156.1	–			
35	Male	20.4	115.2	–			
61	Male	39.2	231	–			
21	Male	45.8	218	–			
27	Male	62.8	298.7	–			
42	Male	63.59	236.6	–			
39	Male	11.9	53	–			
33	Male	94.8	350.6	–			
37	Male	65.3	197.3	–			
27	Male	69	288.3	–			
21	Male	57.9	277.1	–			
40	Male	54.8	233.2	–			
49	Female	63.1	255.8	–			
56	Female	10	58	–			
42	Male	95.5	336.5	Septic			
17	Female	64.6	243	Septic			
17	Female	88.4	324	Septic			
39	Male	53.9	202.4	–			
32	Male	4.8	38	–			
52	Male	95.4	339.9	–			
51	Male	37.3	149.3	–			
56	Female	101.1	339.2				
28	Male	34.3	136				
32	Female	63	248.9				
44	Male	79.4	277.7				
57	Female	52.9	221.8				
57	Female	65.9	236.2				
61	Male	51.2	181.2				
41	Male	105	51.5				
31	Male	24.1	160.6				
51	Male	36.2	180.5				
42	Male	70	309.2				
48	Male	35.4	168.8				
44	Male	80.8	338.5				
38	Female	27	165.8				
54	Female	30.1	218.2				
6	Male	25.1	179.9				
32	Male	31.3	184.3				
41	Male	91.1	412.9				
45	Female	72.7	332.5				
56	Female	73.7	317.1				
45	Male	69.3	283.1				
30	Female	34	140.5				
65	Male	39.2	231				
29	Male	12.5	81.4				
22	Male	10	82.3				
23	Male	21.3	165.8	–			

## Discussion

Cranioplasty serves to protect the previously exposed neural structures and provides patients with cosmetic satisfaction. In addition, it improves the neuropsychological outcome. Studies show that earlier defect restoration results in progressive improvement in patients’ function^7^. Not only can the circulation return to normal conditions, but the cerebrospinal fluid circulation also recovers. Different techniques and materials have been used since ancient times to fulfil those purposes and have had various results^8,9^. From the technical side, neurosurgeons are expecting constant availability, reproducibility and easy handling in the operating room. From the patient side, the implanted material is the most important factor regarding the outcome.

The autograft appears to be the best material for cranioplasty, but in open craniocerebral injuries, it is not possible to spare the bone. Moreover, temporal subcutaneous implantation can lead to substantial resorption. Deep freezing might overcome that issue, but after reimplantation, resorption is still noted^10^.

Metal implants have advantages in terms of durability and infection control. They are precise, reliable, and suitable for everyday routine. Metal printing processes can be performed only in special laboratories at an extremely high cost. Direct fabrication of the implant can be performed by various techniques, such as selective laser or electron beam melting, but this method is practically available only for a few selected institutes^11^.

Custom prostheses made from hydroxyapatite have a low complication rate. The cancellous ultrastructure resembles a diploe, but the high cost restricts their use in general^6^.

Klammert et al.^12^, in their cadaver pilot experiments, used calcium phosphate for powder-based 3D printing that could, in principle, be suitable for direct implantation, but due to infection problems, the use of this method in living organisms is not yet feasible.

Kim et al.^4^ printed a special mould coated with plastic, allowing the fabrication of replacement during surgery; however, applying that coating on the mould and securing asepsis can be complicated.

Small defects with hidden locations, e.g., after neurovascular decompression from the retromastoid approach, can be filled with manual moulding by administering PMMA. The custom-made techniques allow more precise replacement for more extended areas and in more problematic locations. 3D planning and preoperative printing simply disclose the possibility of poor fitting^5,6,12^. No cactus effect, such as when mesh is implanted^8,9^, is possible. Any mismatch occurs at the surface, and the edges can be easily blurred. The implant can be fixed with miniplates or any commercially available devices.

The heat that is released during the polymerization of the PMMA warms the silicone, thus completely eliminating the risk of harmful effects of the heat production in the affected region. If any technical problem arises, implant fabrication can be repeated during surgery, and if the mould is kept, it can be reused for replantation if necessary. Another advantage of polymerizing on a mould is that fewer residual monomers will contact the dura mater directly.

The technique of the 3D-printing based custom-made cranioplasty using silicone moulding with PMMA is easy and reliable. More silicone moulding studies may be explored in further studies. Thin slice CT scans are easily obtainable. The computer-assisted design of the implant results in excellent cosmetics. Planning includes symmetrical mirroring across the midsagittal plane of the skull and follows the contours of the exposed bone. The 3D printing of the whole skull with the prefabricated implant allows the cosmetic result to be checked preoperatively.

To cover cranial defects, PMMA is a widely used material in neurosurgery. It is sterile and available in a less viscous form; thus, the least complicated defects can also be filled with it. The 3D-printed silicone mould can easily be sterilised in a standard fashion in hospitals. Quality control is manageable since the sterilisation of preoperatively mixed and produced PMMA implants raises several technical standards that cannot be followed in labs with smaller case caseloads. The operation time is no longer than that when other techniques are used, since the moulding procedure needs only the original operation time, during which PMMA is expected to solidify, and the procedure can be done by a nurse.

The slightly rough surface of PMMA results perfect adhesion to the skin. Complications may occur by several factors, but skull replacement does not increase the risk.^13–17^. To prevent surgical complications, general principles should be followed. To prevent further complications, the bony edges should be exposed completely since the implant is planned accordingly. It is very important that the skin should be handled with respect. During the first intervention decompression should also be preplanned, presuming that the patient will need reconstruction of the skull^18^. In our cases, we meticulously paid attention to infection prevention. If the skin was broken or presumably weak, we postponed the operation. As an institutional standard, cranioplasty is generally indicated 3 months after craniectomy. Patients receive a bolus of preventive intravenous antibiotics at the induction of the anaesthesia that can be repeated after 4 h if needed to prevent infection. If cranioplasty is presumed, closure of the skin is performed with monofilament materials. The closure of the dura can be a problem in open trauma cases, and in decompressive craniectomy, the dura should be left open as a rule. During cranioplasty, the newly formed encephalomyosynangioses should be preserved as much as possible to prevent bleeding, CSF leakage or delay of expected recovery in already diminished cortical functions. If we pay attention and eliminate all the possible known factors that can lead to complications, the hazard of any operation, such as cranioplasty, can be minimized.

The technique can be used as a secondary option, e.g., in cases where conventional cranioplasty has been performed with complications. In-home sterilisation of the silicone mould and intraoperative preparation of PMMA from the original packaging both minimize the risk of infection. We have not yet used the technique in the paediatric population, but the results are encouraging for implementation.

## Data Availability

All institutions involved in those interventions meets the GDPR data management and archiving guidelines. All of the requested raw datas like anonymized CT scans and 3D models available from the corresponding author on reasonable request.
